# Extended-pulsed fidaxomicin versus vancomycin in patients 60 years and older with *Clostridium difficile* infection: cost-effectiveness analysis in Spain

**DOI:** 10.1007/s10096-019-03503-4

**Published:** 2019-04-13

**Authors:** Carlos Rubio-Terrés, José María Aguado, Benito Almirante, Javier Cobo, Santiago Grau, Miguel Salavert, Elena González Antona Sánchez, Cristina López Gutiérrez, Darío Rubio-Rodríguez

**Affiliations:** 1 Health Value, C/ Virgen de Aránzazu, 21. 5° B, 28034 Madrid, Spain; 2 Department of Infectious Diseases, Hospital Universitario 12 de Octubre, Madrid, Spain; 3 Department of Infectious Diseases, Hospital Universitario Vall d’Hebron, Barcelona, Spain; 4 Department of Infectious Diseases, Hospital Universitario Ramón y Cajal/IRYCIS, Madrid, Spain; 5 Department of Pharmacy, Hospital del Mar, Barcelona, Spain; 6 Department of Infectious Diseases, Hospital Universitario La Fe, Valencia, Spain; 7 Astellas Pharma S.A., Madrid, Spain

**Keywords:** *Clostridium difficile* infection, Cost-effectiveness, Extended-pulsed fidaxomicin, Fidaxomicin, Vancomycin

## Abstract

The cost of treating *Clostridium difficile* infection (CDI) in Spain is substantial. Findings from the randomised, controlled, open-label, phase 3b/4 EXTEND study showed that an extended-pulsed fidaxomicin (EPFX) regimen was associated with improved sustained clinical cure and reduced recurrence of CDI versus vancomycin in patients aged 60 years and older. We assessed the cost-effectiveness of EPFX versus vancomycin for the treatment of CDI in patients aged 60 years and older from the perspective of the National Health System (NHS) in Spain. We used a Markov model with six health states and 1-year time horizon. Health resources, their unit costs and utilities were based on published sources. Key efficacy data and transition probabilities were obtained from the EXTEND study and published sources. A panel of Spanish clinical experts validated all model assumptions. In the analysis, 0.638 and 0.594 quality-adjusted life years (QALYs) per patient were obtained with EPFX and vancomycin, respectively, with a gain of 0.044 QALYs with EPFX. The cost per patient treated with EPFX and vancomycin was estimated to be €10,046 and €10,693, respectively, with a saving of €647 per patient treated with EPFX. For willingness-to-pay thresholds of €20,000, €25,000 and €30,000 per QALY gained, the probability that EPFX was the most cost-effective treatment was 99.3%, 99.5% and 99.9%, respectively. According to our economic model and the assumptions based on the Spanish NHS, EPFX is cost-effective compared with vancomycin for the first-line treatment of CDI in patients aged 60 years and older.

**Electronic supplementary material:**

The online version of this article (10.1007/s10096-019-03503-4) contains supplementary material, which is available to authorized users.

## Introduction

*Clostridium difficile* is the most frequent bacterial cause of hospital-acquired diarrhoea [1]. The annual incidence of CDI in Spain is estimated at 17.1 cases per 10,000 hospitalised patients [2], ranging from 12.2 to 24.0 cases per 10,000 hospitalisations [3, 4]. A study from the United Kingdom found that the rate of hospital mortality is much higher for patients with hospital-acquired CDI (15.3%) than for those without CDI (1.9%), with infection also substantially increasing the length of stay [5].

One study estimated that 7600 episodes of CDI occurred annually in Spain, with an economic burden of €32,157,093 to the National Health System (NHS) [6]. A study from the USA determined that recurrent CDI was associated with significantly greater likelihood of readmission to hospital (85% vs 41%, respectively; *p* < 0.001) and longer length of stay when readmitted (mean 18.6 vs 7.6, respectively; *p* < 0.001) than those patients without recurrent CDI [7]. A recent economic review of published studies reporting CDI-associated burden revealed that in Spain, hospitalisation costs attributable to CDI among all patients were €4265 per patient, rising to €4885 per patient among those aged > 65 years [8]. Furthermore, there was an incremental rise in the cost of treating an initial CDI episode (€3901), first recurrence (€4875) and second recurrence (€5916), with hospitalisation accounting for 96% of costs [8].

Patients with CDI should be managed by discontinuing any antibiotic that might have affected the normal microbial ecology of the large intestine and the use of which favours the proliferation of *C. difficile*, which releases toxins that induce an inflammatory response [1, 9]. Guideline-recommended antibiotic treatments for initial, non-severe CDI include fidaxomicin, vancomycin [10] or metronidazole [11]. However, recurrent infection is common, occurring in up to 25% of cases treated with vancomycin or metronidazole [12]. Recurrence may be due, among other reasons, to delayed recovery of the intestinal microbiota previously disrupted by CDI-directed treatment [13].

Fidaxomicin is a narrow spectrum macrocyclic antibiotic indicated for the treatment of CDI in adults at a dose of 200 mg twice daily for 10 days [11, 14] and has been associated with greater preservation of the intestinal microbiota than vancomycin [15]. Fidaxomicin treatment also significantly lowers the incidence of recurrent CDI compared with vancomycin [16–18]. A validated in vitro human gut model showed that an extended-pulsed fidaxomicin (EPFX) regimen enables the persistence of fidaxomicin at concentrations inhibitory to *C. difficile*, facilitating intestinal microbiota recovery [19]. The efficacy and safety of the EPFX regimen (200 mg oral fidaxomicin twice daily on days 1–5, followed by once-daily administration on alternate days on days 7–25), which uses the same number of tablets as the standard fidaxomicin regimen, were compared with standard vancomycin (125 mg orally, four times daily on days 1–10) in the EXTEND randomised, controlled trial of patients 60 years and older with CDI [20]. The primary endpoint of sustained clinical cure rate 30 days after the end of treatment (day 55 for EPFX and day 40 for vancomycin; defined as clinical response at test of cure and no recurrence of CDI) was significantly higher with EPFX (70%) compared with vancomycin (59%; *p* = 0.030). Until day 90, the rate of sustained clinical cure was significantly higher and recurrence was significantly lower in the EPFX than the vancomycin arm (*p* ≤ 0.007 and *p* ≤ 0.001, respectively) [20]. An economic analysis of these data found that the reduced recurrence rate with EPFX made this regimen more cost-effective than vancomycin for first-line treatment of CDI in older patients from the perspective of the United Kingdom NHS [21].

The objective of the present study was to evaluate the cost-effectiveness of EPFX compared with vancomycin for the treatment of CDI in patients aged 60 years and older from the perspective of the NHS in Spain.

## Methods

### Model design

**Fig. 1:**
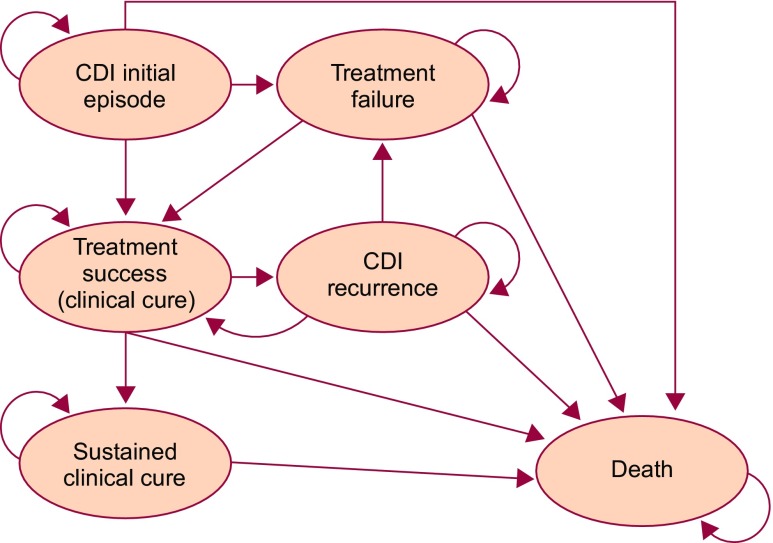
Overview of clinical pathways and health states used in the Markov model. CDI, *Clostridium difficile* infection. Reproduced with kind permission from Watt M, McCrea C. Cost-effectiveness of extended-pulsed fidaxomicin versus vancomycin in older patients with *Clostridium difficile* infection in England. Poster presented at the 27th European Congress of Clinical Microbiology and Infectious Diseases 2017, 22–25 April, Vienna, Austria

Evaluation of the cost-effectiveness of CDI therapy encompasses appraisal of the initial episode and subsequent treatments for CDI recurrence(s). A cohort-based Markov model [22, 23] consisting of six mutually exclusive health states (Fig. 1) was used, evaluating up to three treatment courses. The characteristics of this model have been described previously in an adaptation to the NHS in England [21]. Briefly, the cohort of patients with CDI moved between health states, according to transition probabilities obtained from clinical studies, in discrete periods of time called cycles (every 5 days in the model). During each cycle patients moved between the health states, generating costs and utilities, which represented quality of life on a standard scale of 0 (dead) to 1 (full health), allowing quality-adjusted life years (QALYs) to be estimated, during a time horizon of 1 year [24].

In accordance with the EXTEND study, our model assumed that patients aged 60 years and older with CDI received treatment with EPFX (200 mg oral fidaxomicin tablets twice daily on days 1–5, followed by once-daily administration on alternate days on days 7–25) or vancomycin (125 mg oral capsules, four-times daily on days 1–10) [20]. In our analysis, the hypothetical patient cohort entered the model with an initial CDI episode and received first-line treatment with EPFX or vancomycin (Fig. 1). Further management assumptions are described in Online Resource 1.

Owing to the paucity of information from clinical practice in Spain regarding the choice of therapy for repeated CDI recurrences and for second- or third-line treatment of CDI, treatment choice was estimated by the panel of clinical experts (authors JMA, BA, JC, SG, MS; Table 1). In this regard, several assumptions were made in the model, which are described in Online Resource 1 [10, 23].

**Table 1:** Treatment sequences considered in the model

	First treatment	Treatment received upon first treatment failure	Treatment received upon second treatment failure
Interventional scenario			
Initial CDI episode	EPFX	Tapered vancomycin^a^	FDX 10 days^b^
First CDI recurrence	High-dose vancomycin^c^	EPFX	Tapered vancomycin^a^
Second CDI recurrence	FMT	EPFX	FMT
Comparator scenario			
Initial CDI episode	Vancomycin 10 days^d^	Tapered vancomycin	EPFX
First CDI recurrence	EPFX	Tapered vancomycin	FDX 10 days
Second CDI recurrence	FMT	EPFX	FMT

*CDI Clostridium difficile* infection, *EPFX* extended-pulsed fidaxomicin, *FDX* fidaxomicin, *FMT* faecal microbiota transplantation

^a^Tapered vancomycin regimen: 125 mg four times daily oral vancomycin for 14 days, followed by 125 mg twice-daily oral vancomycin for 7 days, followed by 125 mg once-daily oral vancomycin for 7 days and finally, 125 mg oral vancomycin every 3 days, giving a total of 8 weeks of treatment

^b^FDX 10 days: standard-regimen fidaxomicin consisting of 200 mg twice daily for 10 days

^c^High-dose vancomycin: dose increased to 250 mg four-times daily for 10 days in 80% of patients and up to 500 mg four times daily in 20% of patients

^d^Vancomycin 10 days: standard-regimen vancomycin consisting of 125 mg four times daily for 10 days

### Model inputs

#### Clinical

Comparative clinical and safety data for the EPFX and vancomycin regimens in the treatment of CDI, as well as the model’s main transition probabilities, including death, were obtained from the EXTEND clinical trial [20, 21] (Table 2). Detailed descriptions of other clinical inputs are in Online Resource 2. All clinical inputs were assumed to be the same for the first, second and third treatment courses [21].

**Table 2:** Summary of clinical inputs (cure, recurrence), adverse events and mortality included in the model

Item	EPFX	Vancomycin
Clinical cure, % [20]	*N* = 177	*N* = 179
Clinical cure 2 days after EOT	78.0	82.1
Risk of recurrence, % [20]	*N* = 138	*N* = 147
Recurrence at day 40	1.4	19.7
Recurrence at day 55	4.3	21.1
Recurrence at day 90	7.2	22.4
Incidence of adverse events, % [20]	*N* = 181	*N* = 181
Anaemia	2.8	5.5
Heart failure	2.2	5.5
Constipation	5.5	2.8
Diarrhoea	5.5	6.6
Fever	3.9	6.6
CDI	3.9	13.3
Pneumonia	2.8	5.5
Sepsis	0.6	5.0
Urinary tract infection	3.3	6.6
Mortality, % [20]	*N* = 183	*N* = 181
Days 0–10	1.4	
Days 11–15	1.3	
Days 16–25	1.2	
Days 26–30	1.0	
Days 31–90	0.9	
After day 90	0	

*EPFX* extended-pulsed fidaxomicin, *EOT* end of treatment, *CDI Clostridium difficile* infection

#### Costs

Healthcare resources and the corresponding unit costs considered in the model were obtained from Spanish sources (see Online Resource 3; Table ESM 1) [6, 21, 23, 25–27]. Detailed information on cost inputs are available in Online Resource 3. The analysis was performed from the perspective of the NHS in Spain, and only direct healthcare costs expressed in Euros (€) from 2017 were considered.

#### Utilities

QALYs were calculated from CDI-associated utilities (the quantification of patient-perceived quality of life), obtained from previous studies by Wilcox et al. [28] and Slobogean et al. [29]. The loss of utilities related to adverse events was obtained from several published studies [30–33] (see Online Resource 3, Table ESM 1).

### Model outputs and sensitivity analyses

Results are presented as an incremental cost-effectiveness ratio (ICER), i.e., the cost of gaining one QALY with EPFX compared with vancomycin. A base case (deterministic) analysis was performed, incorporating the mean values of all variables. Several sensitivity analyses were also performed: the duration of hospital stay attributable to CDI was modified, taking the 4 days estimated by Monge et al. [34] into consideration; the impact of outlying values of all the model’s variables was assessed; and finally, the cost of an episode of CDI recurrence was considered to be three times greater than that of an initial episode of CDI [21]. The impact of different probabilities for utilities and costs was assessed using 1000 Monte Carlo simulations [35]. Three willingness-to-pay thresholds were considered: €20,000, €25,000 and €30,000 per QALY achieved with EPFX compared with vancomycin [36, 37].

## Results

### Base case

Applying the EXTEND study outcomes of clinical response to CDI treatment and recurrence (Tables 2 and ESM 1 and Guery et al. [20]) in the model over a time horizon of 1 year, the base case (deterministic) analysis showed 0.638 and 0.594 QALYs per patient treated with EPFX and vancomycin, respectively, a gain of 0.044 QALYs with EPFX (Table 3). Over a 1-year time horizon, the associated treatment cost per patient was €10,046 with EPFX and €10,693 with vancomycin. This would result in a cost saving of €647 per patient treated with EPFX compared with vancomycin. Consequently, EPFX was the dominant treatment associated with higher QALY and lower cost compared with vancomycin (Table 3).

**Table 3:** Results (per patient) of the base case (deterministic) and probabilistic analyses

Base case (deterministic) analysis					
Treatment	Cost	QALY	Cost difference	QALY difference	ICER
EPFX	€10,046	0.638	− €647	0.044	EPFX dominates
Vancomycin	€10,693	0.594			
Probabilistic analysis					
Treatment	Cost	QALY	Cost difference	QALY difference	ICER
EPFX	€10,051	0.635	− €646	0.043	EPFX dominates
Vancomycin	€10,697	0.592			

*QALY* quality-adjusted life-years, *EPFX* extended-pulsed fidaxomicin, *ICER* incremental cost-effectiveness ratio (cost per QALY gained with the most effective treatment)

### Deterministic sensitivity analysis

Sensitivity analyses (Online Resource 4, Table ESM 2) showed that the variables with the greatest influence on outcome were the relative probability of clinical cure with first-line treatment EPFX compared with vancomycin, hospitalisation costs associated with EPFX treatment (days 5–10), the relative risk of recurrence with EPFX compared with vancomycin after 90 days and the utility associated with sustained clinical cure after first recurrence. In all scenarios, EPFX was the dominant treatment versus vancomycin (Online Resource 4, Table ESM 2). Taking into consideration the CDI-attributable hospital stay of 4 days, the sensitivity analysis showed that EPFX was dominant over vancomycin. Given that the cost of a recurrent CDI episode is three times higher than that of an initial episode, the cost per QALY gained with EPFX was €2747 versus vancomycin; therefore, EPFX would be cost effective.

### Probabilistic sensitivity analysis

In the probabilistic analysis, EPFX and vancomycin were associated with costs of €10,051 and €10,697, respectively, with a cost saving of €646 per patient treated with EPFX. EPFX was therefore the dominant treatment (Table 3).

This analysis showed that administration of EPFX had 99.3%, 99.5% and 99.9% respective probabilities of being cost-effective compared with vancomycin at willingness-to-pay thresholds of €20,000, €25,000 and €30,000 per QALY gained. EPFX was therefore dominant over vancomycin.

## Discussion

Our analysis of the treatment of CDI in patients aged 60 years and older revealed that the EPFX regimen was associated with a gain of 0.044 QALYs and a cost saving of €647 per patient compared with vancomycin. The EPFX regimen is a cost-effective treatment in most of the comparative analyses with vancomycin using the range of willingness-to-pay thresholds previously suggested for the NHS in Spain [36, 37].

Any evaluation of our results must take into account both the strengths and potential limitations. Regarding the limitations, it should be borne in mind that this is a theoretical model which is, by definition, a simplified simulation of reality. Also, assumptions had to be made in the model with respect to second- and third-line treatment sequences, and with regard to recurrences, as there was no follow-up of patients who failed to respond to the initial treatment in the EXTEND study [20]. In our model, FMT was the third-line treatment, although this practice is not widespread in Spain, highlighting the differences between clinical practice and recommendations in local, national and international treatment guidelines. However, this was regarded as the superior option as it is recommended by ESCMID and IDSA in the case of multi-recurrent CDI [10, 11]. Owing to the need to complete the model in a way that was fair to both treatment options, two assumptions were applied with regard to third-line treatment: (i) clinical cure would occur in all cases and (ii) there would be no further recurrences. These assumptions were validated by the five clinical experts (authors JMA, BA, JC, SG, MS). Moreover, the same mortality rate was assumed regardless of whether patients received EPFX or vancomycin. In the EXTEND study, one treatment-related death occurred in a patient in the vancomycin treatment arm [20]. The costs associated with recurrent episodes of CDI were assumed to be the same as those for the initial episode. This assumption was conservative, as recurrent CDI episodes can incur higher hospitalisation costs compared with initial episodes in clinical practice [38], although a recent study that estimated resource utilisation for the treatment of initial and recurrent CDI was contradictory, finding higher treatment costs for initial compared with recurrent CDI episodes [39].

The state utilities were not obtained from the EXTEND study, but rather from two published studies: one in patients with CDI, including recurrent CDI [28], and the other in patients with CDI who developed infected wounds following surgery for bone fractures [29]. Regarding the validity of performing our model from a Spanish healthcare perspective and sourcing utility data from other countries, it is notable that in a study based on 83,000 assessments of 44 EQ-5D health states from six European countries, including Spain, there was greater variability between individuals than between countries [40]. All the costs used in our model were taken from Spanish sources [6, 25–27].

The conservative nature of our model did not allow for the inclusion of potential costs associated with reducing the risk of transmission of infection, patient isolation and infection control measures, such as the use of disposable gloves and gowns. As has previously been suggested for standard-regimen fidaxomicin [23], had these costs been included in our model, the results are likely to have been even more favourable for EPFX owing to the lower recurrence rate compared with vancomycin.

The structure of the current model is the same as the one used in a recently published study from the United Kingdom [21]. As in our analysis, the study conducted in the United Kingdom concluded that the EPFX regimen would be the dominant treatment—both cost-saving and more effective than vancomycin [21]—with the cost of a treatment cycle of vancomycin in the United Kingdom being considerably higher than the current regimen in Spain (€214 and €34.50, respectively) [21, 25]. The probability that first-line EPFX was cost-effective at a willingness-to-pay threshold of £30,000/QALY was 76% for the patients in the United Kingdom [21].

The EXTEND study demonstrated sustained clinical cure of CDI and significantly lower recurrence rates with EPFX than with vancomycin in a population of patients aged 60 years and older [20]. The results of this economic model suggest that first-line treatment with EPFX would be cost-effective compared with vancomycin according to the willingness-to-pay thresholds normally considered in Spain.

## Electronic supplementary material

ESM 1 (DOC 240 kb)

## Data Availability

Access to anonymised individual patient level data will not be provided for this trial as it meets one or more of the exceptions described under the Sponsor Specific Information for Astellas on www.clinicalstudydatarequest.com.
