# Supplementary material for: Extended-pulsed fidaxomicin versus vancomycin in patients 60 years and older with Clostridium difficile infection: cost-effectiveness analysis in Spain
Source: Eur J Clin Microbiol Infect Dis. 2019 Apr 13;38(6):1105–11. doi: 10.1007/s10096-019-03503-4 (PMC6520320; doi:10.1007/s10096-019-03503-4)
Supplement: Supplementary file 1 — (DOC 240 kb) [file 10096_2019_3503_MOESM1_ESM.doc]

**Title: Extended-pulsed fidaxomicin versus vancomycin in patients 60 years and older with *Clostridium difficile* infection: cost-effectiveness analysis in Spain**

**Authors:**

Carlos Rubio-Terrés^a^, José María Aguado^b^, Benito Almirante^c^, Javier Cobo^d^, Santiago Grau^e^, Miguel Salavert^f^, Elena González Antona Sánchez^g^, Cristina López Gutiérrez^g^, Darío Rubio-Rodríguez^a^,*

*^a^ Health Value, Madrid, Spain*

*^b^ Department of Infectious Diseases, Hospital Universitario 12 de Octubre, Madrid, Spain*

*^c^ Department of Infectious Diseases, Hospital Universitario Vall d´Hebron, Barcelona, Spain*

*^d^ Department of Infectious Diseases, Hospital Universitario Ramón y Cajal/IRYCIS, Madrid, Spain*

*^e^ Department of Pharmacy, Hospital del Mar, Barcelona, Spain*

*^f^ Department of Infectious Diseases, Hospital Universitario La Fe, Valencia, Spain*

*^g^ Astellas Pharma A.S., Madrid, Spain*

***Corresponding author:**

Darío Rubio-Rodríguez

Health Value

C/ Virgen de Aránzazu, 21. 5º B

28034- Madrid

Spain

*E-mail address:* [drubiorodriguez@healthvalue.org](mailto:drubiorodriguez@healthvalue.org)

*Telephone:* +34 600261084

**Target journal:** *Eur J Clin Microbiol Infect Dis*

**Article type:** Original article

**Word count** (excluding abstract, acknowledgements, tables, figures and legends): 2285

**References:** 40

**Electronic Supplementary Material**

**Online Resource 1.** Model assumptions in relation to CDI management.

At the end of the first treatment cycle (25 days for EPFX, 10 days for vancomycin), patients progressed to one of the following states: (i) treatment success (clinical cure); (ii) second-line treatment owing to failure of first-line treatment; or (iii) patient death. The “death” state included patients who died from CDI or CDI-related complications. “Clinical cure” or “treatment failure” states could also arise after second-line treatment. The model assumed that if there were no recurrent episodes of CDI within a 90-day period, patients would proceed from the “treatment success” state to the “sustained clinical cure” state, and could remain in this health state for the remaining cycles or alternatively, proceed to the “death” state (Fig. 1). It was assumed that clinical cure without recurrence of the infection would occur in all patients treated in the third line.

For repeated CDI recurrences and for second- or third-line treatment of CDI, the following assumptions were made in the model: (i) a first recurrence of CDI following EPFX treatment would be treated with high-dose vancomycin (dose increased to 250 mg QID for 10 days in 80% of patients and up to 500 mg QID in 20% of patients) [23] or a first recurrence following vancomycin would be treated with EPFX; (ii) a second recurrence would be treated with faecal microbiota transplantation (FMT), in accordance with the Infectious Diseases Society of America (IDSA) guidelines [10]; (iii) second-line treatment after failure of EPFX or 10-day vancomycin would include a tapered vancomycin regimen (125 mg QID oral vancomycin for 14 days, followed by 125 mg BID oral vancomycin for 7 days, followed by 125 mg OD oral vancomycin for 7 days and finally, 125 mg oral vancomycin every 3 days; a total of 8 weeks of treatment) [23], or EPFX after failure of high-dose vancomycin or FMT; (iv) third-line treatment would be standard-regimen fidaxomicin (200 mg BID for 10 days) following failure of a tapered vancomycin regimen when EPFX was the first-line treatment, and a tapered vancomycin regimen or FMT following EPFX was the second-line treatment (Table 1).

**Online Resource 2.** Clinical inputs included in the health economic model.

Two efficacy outcomes from EXTEND were used in the analysis: clinical response and CDI recurrence. Clinical response, assessed 2 days after end of treatment, was taken directly from the EXTEND findings and evaluated in the modified full analysis set (mFAS), the primary analysis set for efficacy analyses. CDI recurrence rates (at days 40, 55 and 90) were obtained from the subgroup of patients in the mFAS who achieved a clinical response 2 days after end of treatment [21]. The rates of clinical response or CDI recurrence for vancomycin were utilised directly; for fidaxomicin, risks relative to vancomycin were derived and applied. The probability of CDI recurrence was transformed to a 5 day probability to reflect the cycle length of the model; the probability of clinical cure was applied at end of treatment [21]. Two safety outcomes from EXTEND were utilised in the analysis: incidence of all-grade treatment-emergent adverse events (AEs) reported in ≥5% of patients, and all-cause mortality rates up to 90 days post-randomisation, which included CDI-attributable deaths. For disease-free patients beyond day 90, mortality was assumed to be 0% [21]. This assumption was made based on the short time horizon (<1 year) and because the risk of mortality for disease-free patients beyond day 90 was expected to be equal between the cohorts.

**Online Resource 3.** Cost inputs included in the health economic model.

**Table ESM1.** Unit costs and utilities considered in the model (€ from 2017).

| **Item** | **Cost** | | | **Reference** |
| --- | --- | --- | --- | --- |
| **COSTS** | | | | |
| **Medicinal products** |  | | |  |
| EPFX (200 mg/25 days of treatment) | €1,387.50 | | | [23,25] |
| Vancomycin (125 mg/10 days of treatment) | €34.50 | | | [23,25] |
| Vancomycin at high doses^a^ | €69.00 | | | [23,25] |
| Tapering regimen of vancomycin^b^ | €74.00 | | | [23,25] |
| Rescue treatment (10 days of treatment) | €1,209.27 | | | [23] |
| Fidaxomicin treatment (10 days of treatment) | €1,387.50 | | | [23,25] |
| **Administration of medicines** |  | | |  |
| EPFX | €0 | | | Assumption |
| Vancomycin | €0 | | | Assumption |
| **Monitoring** |  | | |  |
| EPFX | €0 | | | Assumption |
| Vancomycin | €0 | | | Assumption |
| **Hospitalisation for CDI episode** |  | | |  |
| 1 day | €662.76 | | | [6] |
| Days 0–5 | €3,313.78 | | | [6] |
| Days 5–10 | €3,313.78 | | | [6] |
| **Per episode of adverse events** |  | | |  |
| Anaemia | €427.10 | | | [26] |
| Heart failure | €4,372.25 | | | [27] |
| Constipation | €55 | | | [27] |
| Diarrhoea | €1,225.43 | | | [26] |
| Fever | €1,744 | | | [27] |
| Pneumonia | €4,161.25 | | | [27] |
| Sepsis | €6,102.50 | | | [27] |
| **UTILITIES** | | | | |
| **Markov state utilities** | Initial CDI episode | First CDI recurrence | Second CDI recurrence |  |
| CDI; first-line treatment (first 10 days) | 0.42 | 0.30 | 0.27 | [28,29] |
| CDI; second-line treatment (first 10 days) | 0.42 | 0.30 | 0.27 | [28,29] |
| CDI; third-line treatment (first 10 days) | 0.42 | 0.30 | 0.27 | [28,29] |
| Treatment success (clinical cure)/days 10–25 of treatment | 0.78 | 0.56 | 0.56 | [30] |
| Sustained clinical cure | 0.78 | 0.78 | 0.78 | [30] |
| **Loss of utilities per adverse event episode** | | | | |
| Anaemia | -0.08 | | | [30] |
| Heart failure | -0.11 | | | [31] |
| Constipation | -0.01 | | | [30] |
| Diarrhoea | -0.01 | | | [30] |
| Fever | 0 | | | [30] |
| CDI | 0 | | | [30] |
| Pneumonia | -0.01 | | | [32] |
| Sepsis | -0.17 | | | [33] |
| Urinary tract infection | 0 | | | [31] |

EPFX: extended-pulsed fidaxomicin; CDI: *Clostridium difficile* infection.
^a^It is assumed that the dose of vancomycin is increased from the usual 125 mg four-times daily (QID) to 250 mg QID for 10 days in 80% of patients and up to 500 mg QID for 10 days in the remaining 20% [23]

^b^125 mg of oral vancomycin QID for 14 days, followed by 125 mg of oral vancomycin twice daily for 7 days, followed by 125 mg of vancomycin once daily for 7 days and finally by 125 mg of oral vancomycin every 3 days (a total of 8 weeks of treatment) [23]

The costs of antibiotic acquisition, hospital admissions and treatment-related AEs were included. Antibiotic acquisition costs were calculated from approved prices in Spain [25]. The unit cost of hospitalisations was obtained from the country’s NHS public healthcare prices [23,26,27] and from a Spanish study [6]. All costs were updated to 2017 according to the consumer price index of Spain. As in a previously published cost-effectiveness model [21], the hospitalisation cost for the initial CDI episode was assumed to be the same regardless of treatment with EPFX or vancomycin (i.e. patients receiving EPFX were discharged to continue treatment at home after a 10-day hospitalisation period). The cost of AEs was obtained from a study by Isla et al. [26] and from the public prices of the diagnosis-related groups [27]. The cost of third line (rescue) treatment (standard-regimen fidaxomicin or FMT) in the base case of the analysis was estimated conservatively as the average of both treatments. The cost of the recurrent episodes of CDI was assumed to be the same as that of the initial episode.

**Online Resource 4.** Results of the deterministic sensitivity analysis.

**Table ESM2.**

| **Parameters** | **ICER (€/QALY)** | |
| --- | --- | --- |
|  | **ICER Lower** | **ICER Higher** |
| Utility (2nd recurrence) CDI; 1L on treatment (first ten days) | EPFX dominates | EPFX dominates |
| Utility (2nd recurrence) Treatment success (clinical cure) / days 10–25 on treatment (EPFX patients only) | EPFX dominates | EPFX dominates |
| Utility (2nd recurrence) Sustained clinical cure | EPFX dominates | EPFX dominates |
| Vancomycin tapering (days 5–10) | EPFX dominates | EPFX dominates |
| Utility (1st recurrence) CDI; 2L on treatment (first ten days) | EPFX dominates | EPFX dominates |
| Utility (initial episode) CDI; 2L on treatment (first ten days) | EPFX dominates | EPFX dominates |
| Utility (1st recurrence) CDI; 3L on treatment (first ten days) | EPFX dominates | EPFX dominates |
| Utility (initial episode) CDI; 3L on treatment (first ten days) | EPFX dominates | EPFX dominates |
| Utility (initial episode) CDI; 1L on treatment (first ten days) | EPFX dominates | EPFX dominates |
| EPFX: recurrence at day 40 (RR vs vancomycin) | EPFX dominates | EPFX dominates |
| Utility (1st recurrence) CDI; 1L on treatment (first ten days) | EPFX dominates | EPFX dominates |
| EPFX: recurrence at day 55 (RR vs vancomycin) | EPFX dominates | EPFX dominates |
| Vancomycin: recurrence at day 55 | EPFX dominates | EPFX dominates |
| Vancomycin: recurrence at day 40 | EPFX dominates | EPFX dominates |
| Vancomycin: clinical cure | EPFX dominates | EPFX dominates |
| Utility (1st recurrence) Treatment success (clinical cure) / days 10–25 on treatment (EPFX patients only) | EPFX dominates | EPFX dominates |
| Utility (initial episode) Treatment success (clinical cure) / days 10–25 on treatment (EPFX patients only) | EPFX dominates | EPFX dominates |
| Rescue treatment Hospitalisation costs (day 0–5) | EPFX dominates | EPFX dominates |
| Utility (1st recurrence) Sustained clinical cure | EPFX dominates | EPFX dominates |
| Utility (initial episode) Sustained clinical cure | EPFX dominates | EPFX dominates |
| EPFX: recurrence at day 90 (RR vs vancomycin) | EPFX dominates | EPFX dominates |
| Vancomycin: recurrence at day 90 | EPFX dominates | EPFX dominates |
| EPFX Hospitalisation costs (day 5–10) | EPFX dominates | EPFX dominates |
| EPFX Hospitalisation costs (day 0–5) | EPFX dominates | EPFX dominates |
| EPFX: clinical cure (RR vs vancomycin) | EPFX dominates | EPFX dominates |
| Vancomycin Hospitalisation costs (day 5–10) | EPFX dominates | EPFX dominates |
| Utility (2nd recurrence) Treatment success (clinical cure) / days 10–25 on treatment (EPFX patients only) | EPFX dominates | EPFX dominates |
| Utility (2nd recurrence) Sustained clinical cure | EPFX dominates | EPFX dominates |
| Vancomycin tapering (days 5–10) | EPFX dominates | EPFX dominates |
| Utility (1st recurrence) CDI; 2L on treatment (first ten days) | EPFX dominates | EPFX dominates |

EPFX: extended-pulsed fidaxomicin; 1L, 2L, 3L: first-, second-, third-line; CDI: *Clostridium difficile* infection; ICER: incremental cost-effectiveness ratio (cost of gaining one QALY with EPFX vs vancomycin); RR: relative risk.
